# Pyrogenum (Pus from Septic Abscess)

**Published:** 1893-08

**Authors:** W. A. Yingling

**Affiliations:** Nonchalanta, Kansas


### PYROGENUM (PUS FROM SEPTIC-ABSCESS).

W. A. Yingling, M. D., Nonchalanta, Kansas.

I here collate the *reliable* indications of *Pyrogenum.* I have not used any items from the work of Dr. Burnett, as that work is obtainable by every one. I have omitted to record any symptom where the action of other remedies used in connection with *Pyrogenum* might influence its curative range. As the larger part of this record is clinical, and as the *symptoms cured with a single remedy* are reliable data, I do not indicate the difference. I use the language of the authors as far as practicable.

Authorities: A proving by G. W. Sherbino, M. D., *Med. Advance,* XXV, 369; M. Florence Taft, M. D., *Med. Advance,* XXV, 378; H. C. Allen, M. D., *Med. Advance,* XXVI, 36; W. H. Leonard, M. D., *Med. Advance,* XXVI, 103; J. A. Wakeman, M. D., *Med. Advance,* XXVIII, 298; G. W. Sherbino, M. D., Homœopathic Physician, XIII, 204; Jennie Medley, M. D., Homœopathic Physician, XIII, 114; J. Emmons, M. D., Homœopathic Physician, XIII, 284; personal letter, Samuel Swan, M. D., J. H. Allen, M. D.

*Mind: Very loquacious.* "I never talked so much in one day in all my life." "I could think faster and talk faster than I ever could." Great desire to talk.

Irritability.

Feels good and buoyant, in the best of spirits, although feels sick.

Delirious when closing the eyes; sees a man at the foot of the bed or in the further part of the room.

Inclined to talk all the time at night during the fever. Talks to herself. Whispers to herself; whispers in her sleep.

Sensation as if she covered the whole bed; she knew that her head was on the pillow, but she could not tell where the rest of her body was.

She feels when lying on one side that she is one person and that when she turns to the other side she is another person.

Sensation as though the fever would not run in her alike—*i. e.*, she felt as though she was existing in a second person, or that there were two of her.

Sensation as though he was crowded with arms and legs; when turning over in bed they were still crowding him.

After the fever leaves him he still has the *hallucination that he is very wealthy*, and that he has a very large sum of money in the bank; this idea of having money was the last to leave him.

*Sensorium:* On getting up in the morning staggers as if drunk.

Dizziness on rising up in bed.

*Head:* Pain in both mastoids, aching worse on right side. A dull thobbing in the mastoid region.

*Great throbbing of the arteries of the temple and head;* every pulsation felt through the brain; this throbbing would meet on top of the brain. Every pulsation felt in the head and ears.

*Painless throbbing:* Throbbing all through the fore part of the head; sounds like steam escaping from a steam tug; a puffing and purring sound.

Forehead bathed in cold perspiration.

Frightful throbbing headache, which was better from a tight band.

In the case of *Bright's Disease* the headaches had most terrible aggravations, lasting from two to four days; during the aggravation she could neither lie in bed nor sit up, but was in constant motion, groaning and crying piteously for help.

Excruciating, bursting, *throbbing headache with intense restlessness.* Headaches were often accompanied with profuse bleeding of the nose and nausea and vomiting.

Sensation as if a cap were on the head; when she awakens and finds this cap on her head she knows she is all right, that she is not delirious.

Cerebro-spinal meningitis. (See Limbs in General.)

Rolling of the head from side to side.

*Eyes:* Left eyeball sore, worse by looking up and turning the eyeball outward.

Projecting eyes.

*Ears:* Loud ringing in left ear, like a bell; also in right ear, lasting a few moments.

Cold ears.

Redness of the ears, looked as if the blood would burst out of them.

*Nose:* Awakened by dreaming that the nose was bleeding, and found it had bled all over the pillow.

Sneezing every time he puts his hand out from under the cover.

Sneezing at night; nostrils closing, alternating from side to side.

Cold nose.

*Fan-like motion of the alæ nasi.*

*Face:* Burning of the face. Face yellow. Face very red. Face pale and sunken and bathed in cold sweat. Face pale, greenish or chlorotic.

Circumscribed redness of the cheeks.

*Tongue and taste:* Tongue coated white on the forepart and brown on the back part. Brownish coat on tongue, mostly back part. Tongue coated a yellowish brown. Tongue heavily coated, yellowish gray fur; edges and tip very red. Yellow brown streak down centre of tongue.

*Terribly fetid taste*, as if mouth and throat were full of pus (which lasted some twenty-four hours; could only associate it with a broken abscess in the mouth. Proving).

*Tongue fiery red, then dark red and intensely dry.*

Tongue clean, smooth and dry; first fiery red and then dark red, glossy, shiny, and easily moistened.

Tongue dry, not a particle of moisture on it. Tongue dry down the centre.

*Mouth: Bad taste in the mouth.* Bad taste in the morning. Terribly fetid taste in the mouth and throat as if full of pus.

Breath horribly offensive, carrion-like.

Bitter taste in the mouth.

*Throat:* Diphtheria with extreme fetor; rotten odor.

*Appetite:* No appetite for breakfast. No appetite for dinner. No appetite nor thirst.

*Eating and drinking:* Great thirst for small quantities, but the smallest quantity was instantly rejected by the stomach.

Drinking very hot water relieves the sick stomach.

Entire absence of thirst.

*Nausea and vomiting:* Belching of sour water after breakfast.

Vomiting and purging; stool profuse and watery.

Nausea and vomiting persistent.

Vomiting water when it becomes warm in the stomach.

Sick stomach better by drinking very hot water.

Vomiting ameliorates the sick stomach.

Tries to vomit; urging to vomit, with cold feet.

Feels better after vomiting.

*Stomach:* Feels too full in the stomach and bowels.

*Abdomen:* Great distention. Feels too full.

Pain in the umbilical region, with passage of sticky, yellow stool.

While riding in a buggy aching in, or pain on, left side of the umbilicus. Aggravated by drinking water. Ameliorated by passing flatus downward.

Soreness of the abdomen. Bowels so sore she can hardly breathe. Bowels so sore she cannot bear any weight or pressure over right side.

*Sides:* Bubbling or gurgling sensation in left hypochondrium, extending back to the left side of the spine; felt only when lying on the left side.

Very severe pain in the right side; knife-like pains going through the back; *worse* from every motion, from coughing, talking, or taking a long breath; *better* from lying on the affected side; groaning with every breath.

*Stool, anus, etc.:* Two soft, sticky stools, eight to nine A. M.

On passing flatus per anus a portion of stool passed involuntarily.

Stool very much constipated, large and difficult; required great effort; first part of stool composed of balls, last part natural; on stool there were streaks of blood, leaving a soreness in anus.

*Constipation,* hard, dry, accumulated feces.

Vomiting and purging; stool profuse and watery; no pain.

*Stool horribly offensive,* a carrion-like odor.

Tenesmus with strong desire to defecate without ability, reflected from the bladder. (See under Urine.)

*Urine:* Bright's disease of the kidneys.

Urine less frequent than usual; only twice in twenty-four hours.

Urine very yellow after being made, scant; do not desire to urinate half as often as in health; urine after standing gets very cloudy with a substance that looks like orange peel; a red line was deposited on the side of the vessel which was hard to remove.

Urine yellow. Urine scant.

Red sidiment at the bottom of the vessel looking like red pepper.

Got up three times to urinate at night.

Urine loaded with albumen, casts frequent, but never numerous.

*Urine horribly offensive, carrion-like.*

As soon as the fever came on he commenced to urinate; could tell when the fewer was coming on from the frequent calls to urinate. This urine was as clear as spring water.

Intolerable tenesmus of the bladder, it was more of a spasmodic contraction than anything else; at the same time this condition would be reflected to the rectum with a strong desire to defecate without ability to do so; involving also the ovaries and the broad ligaments.

The following case will be of interest in this connection. On a former occasion I had given *Catarrh-vesica*$^{dmm}$ (Swan), which acted promptly and removed the distressing symptoms, but they returned in time. I concluded to test the remedy under consideration, with the following result: June 4th, 1893.—A lady of thirty-nine years, blonde. Severe tenesmus of the bladder, involving the vagina and rectum, with soreness of the muscles of the abdomen. "The rectum would draw together so tightly she could not introdnce her finger." She would have urging desire to pass water every few minutes, passing but very little *foul* urine at a time, with severe straining to pass more water. After each evacuation of urine a continual burning and pressure in the parts. Some relief, or rather endurance, by sitting over the urinal, which allowed the passage of the water at all times. There was a comfortable feeling in sitting over hot water. The woman suffered continual agony. At 9.30 A. M. I prescribed *Pyrogen.*$^{cm}$ (Swan), a small powder in eight spoons of water, a spoonful every hour till better. In a very few moments after taking the first spoonful she commenced to gap, and in less than one-half hour she went to sleep and slept for two and one-half hours. A little after noon she got up, feeling much better, and prepared dinner for the family. This work slightly aggravated the symptoms, upon which she took the second spoonful of medicated water and again slept for a couple of hours to awake feeling very much better, and by six P. M. perfectly relieved in every way except a slight hurting in the forehead and small of back; next day was still feeling better, but had a soreness in the region of the kidneys.

June 5th.—Felt quite well all day. Only trouble is a sense of weakness, or a don't-feel-like-doing-anything; in the private parts a sense of fullness, but none of the distressing symptoms of yesterday. When quiet she can feel her "pulse" beat all over the body; when sitting she feels it in her "bottom." No medicine.

June 6th.—"Feels very good this morning, yet feels that she has not entirely gotten rid of the trouble." Slept all night soundly; a good night's rest. Still feels the pulsation, but not so strongly. From too much exercise during the day she began to feel badly in the afternoon, with some strong indications of the trouble returning. She had to "hold up" in the privates and rectum; rectum felt drawn up or contracted, some headache. *Pyrogen.*$^{dmm}$ (Swan), one dose dry on the tongue on going to bed.

June 7th.—Feels better than at any time yet. No headache; the "holding up" and contraction in private parts and rectum all gone. Her abdomen feels sore still, and she is weakly but much encouraged. Remains better all day, but still "feels it to be there yet, but much better." No medicine.

June 8th.—Still improving. Feels the trouble only when passing water, and then only a very little. Feels "lots better in every way," but does not gain strength so rapidly as she would like. No medicine.

June 9th.—Felt some worse last night, which must have been from too much exercise and standing. Feels better this morning. Does not feel the trouble at all. No medicine.

June 10th.—Doing well. No medicine.

June 11th.—From a ride in a poor, shaky cart, which jarred her very much, last night the burning pressure and tenesmus returned so as to be distressing. A single dose of *Pyrogen.*$^{dmm}$ (Swan), at once relieved and produced sleep during the night. Next morning quite well, and no more medicine.

June 14th.—Has been doing finely and without a return of the trouble. Has taken no more of the medicine. To-day, after assisting in a large washing, her menses came on entirely free from pain and of a bright red blood, and feeling unusually well. Other periods have been not to say painful, but disagreeable, with *very dark offensive blood, which could hardly be washed from the napkin.*

June 20th.—Has had on a couple occasions slight indications of a return of the trouble during the evening, but it passed off without any more medicines.

July 11th.—Remains well and feels better than for many months. This is her condition in spite of very much work. (W. A. Y.)

*Male Sexual Organs:* Testicles hang down relaxed; the scrotum feels thin and it looks that way.

*Female Sexual Organs:* Puerperal peritonitis with extreme fetor; a rotten odor.

Genitals seriously swollen. Bright's disease.

*Menses horribly offensive, carrion-like.*

Menses last but one day, and then a sanguineous leucorrhœa of the *horrible odor.*

Hemorrhage of bright red blood with dark clots.

Septicæmia following abortion.

Has cured prolapsus uteri, with bearing down, relieved by holding the breath and straining, as in the act of labor.

Pains start in the uterine region and passing upward to the umbilicus. It was the reverse [in direction] (a corkscrew pain); this was momentarily relieved by holding the breath and bearing down, as in labor; momentarily relieved by pressing the hands against the vulva; then she said she would have to turn loose, as it made her worse.

Pain starting in the umbilicus or a little above and passing down toward the uterus, but at midway of the distance to the uterus it would be intercepted by the same kind of a pain starting in the uterus and passing upward till they would meet midway between the umbilicus and uterus, then gradually die out, till another would come as before; ameliorated momentarily by drawing her knees up to her chin and grasping her arms around them and holding them tight.

In a case of abscess of left ovary following oophoritis, the ovary very large, throbbing, acute pain, great distress, with fever and rigors, the administration of *Pyrogen.*$^{cm}$ (Swan), produced a flow, as reported by the husband, of "white, creamy pus the size of a man's arm from the womb," with general amelioration of the condition and rapid decrease of the size of the ovary. The case was cured, but required *Lachesis* and *Calcarea-carb.* There is no question of the speedy and happy effect of *Pyrogen.* (W. A. Y.).

*Breathing:* Wheezing when expiring.

*Cough:* Coughing, spitting up phlegm from the larynx.

Cough worse from moving, turning over, or the least motion.

Coughs more in a warm room. Cough better by sitting up; worse when lying down.

Coughing up rusty colored mucus. Expectoration horribly offensive. Coughing up yellow sputa through the night.

Burning in the larynx and bronchi on coughing; coughing causes a pain in the back of the head.

Stitches in small of back on coughing, only noticed in the chair.

*Lungs:* Pain in the right lung and shoulder, aggravated from coughing or talking.

Neglected pneumonia: "Cough, night-sweats, frequent pulse, and to all appearance as if in the last stage of pneumonic-consumption. An abscess had burst that day and was discharging a great amount of pus; tasted like matter." Made a rapid recovery on *Pyrogen.*$^{cm}$, three doses.

*Heart and Pulse:* Pain in the region of the left nipple, as if in the heart, as if it was going to ache; increased heart action; pulse 120.

*Every pulsation felt through the brain.*

*Heart feels tired* as after a long run.

Increased heart action from least motion.

Circulation so active he could hear the blood pass through the ears and sound like escaping steam. Blood throbbed all through fore part of head; could feel it in every part of the body, even in the fingers.

*Tired feeling about the heart,* feels like taking it out to let it rest; it would be such a relief to stop it and let it lie down and stop throbbing. Pulse, 96; temperature, 98, nine A. M.

Every pulsation felt in the head and ears; a painless throbbing.

Sensation as if the heart was enlarged; a distinct consciousness of a heart.

Pulse, 140, feeble and wiry; temperature, 99. (Pulse, 160.)

Violent heart action which is very tiresome.

*Palpitation or increased action of the heart without corresponding increase of temperature.*

Palpitation worse from motion, better by remaining quiet.

Heart beats hard, has a laborious action.

Sensation as if the heart was too full of blood.

Heart beats very hard; can be heard a foot away from the thorax.

Always can hear her heart beat.

Could not sleep from the whizzing and purring of the heart; when she did drop off to sleep she was delirious.

*Chest:* "A severe hurting within the lower part of the sternum, sometimes extending to the joints of the ribs, and up to the throat, as if the œsophagus was being cramped; a sensation of contraction, of drawing together."

Soreness of the chest; purple spots on the chest.

*Neck and Back:* Weak feeling in the back.

Stitching pain in the back on coughing; pain in small of back.

Throbbing of the vessels of the neck; the carotids have a distinct wave-like throb. The throbbing is from the clavicle upward.

*Upper Extremities:* Abscess in left shoulder joint, in front; seemed to be in the joint extending down the arm for three inches, the pain lasting till I went to bed. (From five P. M.)

Numbness of the hands and arms.

Hands cold and clammy.

Pain in the right lung and shoulder; worse from coughing or talking.

*Lower Extremities:* Aching about the left knee as though the bones were broken.

Aching about the knees, deep in the bones, while sitting by a fire. Better by walking around and motion and by putting the legs on the stretch.

After going to bed felt a pain about the patella, by flexing the leg.

Tingling sensation on the right little toe as if frost-bitten.

Feet and legs seriously swollen. Φ Bright's Disease.

Legs swollen to utmost extent of skin. Φ Bright's Disease.

Numbness of the feet.

*Limbs in General: Aching pains in the limbs; aching in the bones; aching all over the body* as from a cold; aching with soreness of the flesh; the bed feels hard.

Cold extremities.

Numbness of the hands and arms, and of the feet, and the numbness extends over the whole body.

Child with cerebro-spinal meningitis *very sick;* there was automatic motion of the right arm and right leg; this kept up till it would turn her around from left to right till her feet would get on the pillow or touch the head-board. When righted in bed the same would be repeated at once.

*Fever, Chills:* "In all cases of fever commencing with pains in the limbs." (Swan.)

*Chilliness:* Chilly all night; the bed feels hard.

Feverishness.

Felt chilly, with increased action of the heart.

After getting into bed felt chilly, teeth chattered. Awoke at ten P. M. in a perspiration. Sweating mostly on upper part of the body.

Feels hot as if he had a fever, but the temperature was only 99°; feels as if it was 105°.

*Perspiration horribly offensive,* carrion-like; disgust up to nausea about any effluvia arising from her own body.

Cold sweat over the body.

Coldness and chilliness all day that no fire would warm; sits by the fire and breathes the heat from the stove; chilly whenever leaving the fire; at night when the fever came on he had a sensation as if his lungs were on fire, and that he must have fresh air, which soon brought relief.

Frequent calls to urinate as soon as the fever came on; urine clear as spring water.

*Sleep:* Dreams about various things—*about business*—dreaming all night; "dreaming that three ladies were stopping at the same house; we all had the diarrhœa and were all cured by *Aloe*."

After sleeping awhile awoke to roll and tumble in every conceivable position.

Feels weak in the morning.

Activity of the brain prevents sleep; was making speeches and writing articles; could not keep my eyes shut. Could not sleep till toward dawn.

Restlessness relieved after sleep.

Cries out in her sleep that some one or a weight is lying on her.

Could not sleep from the whizzing and purring of the heart.

Whispers in her sleep.

*Nerves:* Great debility in the morning so that he staggered when trying to walk.

Great nervousness and restlessness.

Could not lie long in one place without moving. "Thought she would break if she laid too long in one position."

Great prostration.

*Great restlessness, better when first commencing to move;* relief is but momentary; must keep up the motion.

Death-like restlessness or the restlessness of death or those in *articulo mortis;* amelioration from sitting up in a chair and rocking hard.

Symptoms of paresis; child could not stand nor walk; sat on the edge of the bed rocking the body back and forth—was relieved when in motion.

Restlessness relieved after sleep.

*Tissues:* Septic states.

Typhoid conditions.

Bones ache.

*Generalities: Aching all over; bed feels hard;* can lie but a few moments in one position; aching with soreness of the flesh.

The rapidity of the pulse, far above the temperature, seems to be a keynote.

"Knew he was going to have typho-malarial fever, which he had two years previously, after a malarious exposure on a foreign mission field. Had every other day what he called 'dumb ague.'" *Pyrogen.* cured.

Relief from heat. Very fond of the hot bath.

*Pyrogen.* has cured several cases of *blood poisoning.* It should be thought of in dissecting wounds.

The hard bed, the hard pillow, and the intense aching that they sometimes compare to lying on a pile of rocks shows the intense soreness of *Pyrogen.;* sometimes the patient declares that a train of cars has run over him.

Aggravation from sitting up in bed; from rising up.

Amelioration of the death-like restlessness from sitting up in chair and rocking hard. Amelioration of cough from sitting up in chair.

In all fever cases when other remedies do not act, think of Pyrogen. (Swan.)

In septic poisoning from wounds, after abortion, accouchement, etc., etc., think of *Pyrogen.* (J. H. Allen.)

*Relationship:* "*Pyrogen.* resembles Arnica, Baptisia, and Rhus in the aching and hard bed. It is more similar to Rhus, as the restlessness is better from changing the position, or motion. The restlessness is as great as in Rhus, and Rhus is an antidote to Pyrogen. The cough is more like Bryonia as it is worse from motion and in a warm room." (Sherbino.)

*Pyrogen.* resembles Ipecac very closely in uterine hemorrhage. If you have an Ipecac case of uterine hemorrhage, and that remedy fails you, don't fail to think of *Pyrogen.*

